# Testing the knowledge of Alzheimer's disease *via* an intervention study among community health service center staff in Jiaxing, China

**DOI:** 10.3389/fpubh.2022.969653

**Published:** 2023-01-27

**Authors:** Weiwei Ma, Liping Zhu, Jiangang Tang, Wanli Diao, Liqi Qian, Xiaoyang Feng, Xiaoling Zhang

**Affiliations:** ^1^Department of Neurology, The Second Affiliated Hospital of Jiaxing University, Jiaxing, China; ^2^Department of General Practice, Jiabei Street Community Health Service Center, Jiaxing, China; ^3^Zhejiang Chinese Medical University, Hangzhou, China

**Keywords:** Alzheimer's disease, community, knowledge, training methods, influencing factors

## Abstract

**Objective:**

This study aimed to investigate the knowledge status of Alzheimer's disease (AD) among community health service center (CHSC) staff in Jiaxing, China, and to compare the effects of online with offline training.

**Methods:**

A total of 763 people from 12 community health service centers were investigated using a self-created general situation questionnaire and the Alzheimer's Disease Knowledge Scale (ADKS). Among the participants, 261 people who were willing to receive training were randomly divided into two groups according to the institution in which they worked to receive online or offline training, respectively.

**Results:**

The average ADKS score was 19.77, and the awareness rate was 65.92%; the results for every field were as follows: treatment and management (81.32%); life impact (77.76%); disease course (75.23%); assessment and diagnosis (68.94%); risk factors (65.05%); symptoms (57.90%); caregiving (44.06%). Education and profession had impacts on the total ADKS scores (*P* < 0.05). A total of 261 people participated in the training, and there were significant differences in ADKS scores before and after training (*P* < 0.05). Before the training, there was no significant difference in ADKS score between the two groups; after the training, either (*P* > 0.05). There were significant differences in the ADKS scores after training in both groups (*P* < 0.05).

**Conclusion:**

Community health service center staff in Jiaxing had limited knowledge of AD, particularly in the “symptom” and “caregiving” dimensions. One instance of training on AD-related knowledge to some degree helped to improve this but still fell short of meeting the national requirements. No significant differences were found between offline and online training effects.

## Background

Alzheimer's disease (AD) is a neurodegenerative condition characterized by progressive cognitive dysfunction and behavioral impairment that occurs in pre-old age and old age (1). The condition is the most common type of dementia, accounting for about 50–70% of cases (2). There were about 47 million dementia patients worldwide in 2015; this number is expected to rise to 131 million by 2050 (3, 4). Huang et al. (5) reported the prevalence of dementia in the elderly aged 65 and above in China as 5.6% (3.5–7.6%), more than 60% of which were AD. The total direct and indirect cost of AD in China is more than ¥1 trillion annually (6), signifying a significant burden for families and society.

The focus on dementia and Alzheimer's disease has continued to increase in recent years. In September 2020, the National Health Commission announced the management of screening for Alzheimer's disease within basic national public healthcare services (National Health Office Disease Control Letter, 2020, No. 726) and instituted the requirement that public awareness rates regarding the prevention and treatment of AD in pilot areas be increased to 80% by 2022. All relevant medical staff in grassroots healthcare services should thus have knowledge about the prevention and treatment of dementia.

According to the Statistical Bulletin of the Elderly Population and Undertakings for the Aged in Zhejiang Province,^1^ the elderly population aged 60 and older was 11,526,100, of which Jiaxing represented 26.68%, ranking second in Zhejiang Province overall. Based on a conservative estimate, there are more than 172,000 patients with AD in this region. However, as one of the first areas in the province to develop its aging population, cognitive disorders such as AD have not yet been added to chronic disease management, and there are no community screening or training programs in place based on detecting the disease. The purpose of the present study was thus to investigate the current knowledge on AD among community health service center (CHSC) staff in this region, to provide targeted training, and to evaluate the training effect to provide a basis for further training and government decision-making.

The ADKS is applicable to AD patients and their caregivers, elderly people in the community, students, and medical staff and has high reliability and validity (7). Garcia-ribas et al. (8) explored ADKS-item characteristics using item response theory procedures and found that although the ADKS did not present a unidimensional structure, its independent items together provided a comprehensive spectrum of information regarding AD knowledge. The Chinese version of the ADKS was translated by the team of He, who showed that it also had good reliability and validity, and was suitable for measuring the AD knowledge of patients and caregivers, students, and medical staff with a Chinese cultural background (9).

## Method

### Sample and settings

Jiaxing includes three districts, as well as two county and three county-level cities, each with 4–12 streets or towns. From May to November 2020, 12 representative streets/towns were randomly selected by stratification according to geographical location and size. Following on, all the staff of community health service centers in these streets/towns was selected. A total of 860 questionnaires were returned; 97 were excluded for being too casual in nature; e.g., their answers were all “correct” or all “wrong,” the total time taken to complete them was very short, and questionnaires were repeated or had illegible handwriting. Finally, 763 valid questionnaires were collected. Among these, 261 individuals volunteered to participate in AD-related knowledge training and were randomly divided into an offline (142) and an online (119) training group, based on the institution in which they worked.

The study's inclusion criteria were as follows: (1) staff who had worked in community health service centers for more than 1 month as of March 1, 2020; staff who had good compliance, and were willing to cooperate with the investigation and training.

The study's exclusion criteria were as follows: (1) staff from community health service centers who treated AD and other neuropsychiatric diseases; (2) staff who had poor compliance and refused to cooperate with the investigators.

All of the participants were informed about the research purpose, agreed to participate in the study, and actively cooperated with the investigation and training.

### Survey instruments

A self-designed questionnaire for collecting general information, including name, gender, age, institution of employment, educational background, professional title, and current professions years of employment, whether any previous training related to AD had been received in the past, and whether participants' relatives/friends suffered from dementia or AD.

The Alzheimer's Disease Knowledge Scale (ADKS), which comprises 30 true/false items, includes the following seven dimensions: risk factors (six items), assessment and diagnosis (four items), symptoms (four items), disease course (four items), life impact (three items), caregiving (five items), and treatment and management (four items). The total score ranges from 0 (worst) to 30 (best).

### Data collection and quality control

#### Questionnaire survey

Before and immediately after completing the training, the self-designed questionnaire and ADKS were completed in two ways; the offline training group filled in paper, and the online training group completed the questionnaire digitally using a provided link. Additionally, the online training group was invited to evaluate and provide feedback about the training they received. None of the participants were able to provide the correct answers after completing the ADKS for the first time.

#### Training methods

The offline training group engaged in traditional learning, i.e., by gathering in a specific area where a lecturer presented a slide presentation and content explanations. For the online training group, the lecturer pre-recorded the training information and uploaded it to either the DingTalk or WeChat platforms. The members of this group finished the training on their own within the specified time. The content of the course was the same for both groups and the training duration was 1 h.

Two neurology physicians were uniformly trained and qualified prior to conducting the survey. Following on, they conducted the questionnaire survey, and the AD-related knowledge training, and completed the data collection. The quality was controlled by at least one chief neurology physician.

### Statistical analysis

Data were input into Microsoft Excel 2019, and the SPSS Statistics 25.0 software program was used to conduct statistical analysis. Quantitative data were presented in the form of median and interquartile spacing, which did not conform to normal distribution; a rank-sum test was conducted for making comparisons between the groups. Qualitative data were expressed as the number of cases (percentage), and a rank-sum test was used for comparison between groups in this context. The influencing factors were analyzed by multi-factor linear regression analysis. Statistical significance was based on a *P*-value of <0.05 in two-tailed tests.

## Results

### Participant characteristics

A total of 763 individuals from 12 community health service centers participated in the study. Most of the participants were female (73.0%), with an average age of 35.09 years; most had a bachelor's degree (71.6%), as well as junior or intermediate titles (74.2%), and 49.0% had majored in internal medicine. Their average employed time was 12.87 years. The majority reported that they had never received any training on AD-related knowledge (71.2%), and they had no relatives or friends who suffered from dementia or AD (80.5%). The sociodemographic characteristics of the sample are shown in Table 1.

**Table 1:** Sociodemographic characteristics of the sample.

**Characteristics**	***n*** **(%)**	**Median (quartile distance)**
**Gender**		
Male	206 (27.0%)	
Female	557 (73.0%)	
**Age**		34.00 (13.0)
20–29 years old	243 (31.9%)	
30–39 years old	294 (38.5%)	
40–49 years old	176 (23.1%)	
50–59 years old	44 (5.8%)	
60–69 years old	6 (0.8%)	
**Education**		
Secondary	33 (4.3%)	
Vocational training	179 (23.5%)	
Bachelor	546 (71.6%)	
Master and above	5 (0.7%)	
**Professional title**		
Junior	358 (46.9%)	
Intermediate	208 (27.3%)	
Associate senior	64 (8.4%)	
Senior	17 (2.2%)	
Others	116 (15.2%)	
**Profession**		
Physician	374 (49.0%)	
Surgeon	35 (4.6%)	
Nursing	195 (25.6%)	
Pharmacy	56 (7.3%)	
Administration	55 (7.2%)	
Support	38 (5.0%)	
Technician	10 (1.3%)	
**Working years**		11.00 (14.0)
0–10 years	313 (41.0%)	
10–20 years	263 (34.5%)	
20–30 years	140 (18.4%)	
30–40 years	38 (5.0%)	
40–50 years	6 (0.8%)	
50–60 years	3 (0.4%)	
**Training on AD knowledge**		
Yes	220 (28.8%)	
No	543 (71.2%)	
**Relatives or friends had dementia or AD**		
Yes	149 (19.5%)	
No	614 (80.5%)	

AD, Alzheimer's disease.

### Alzheimer's Disease Knowledge Scale scores

The average ADKS score of 763 staff members was 19.77, and the awareness rate was 65.92%. The awareness rate of each specific field (from high to low) was as follows: treatment and management (81.32%); life impact (77.76%); disease course (75.23%); assessment and diagnosis (68.94%); risk factors (65.05%); symptoms (57.90%); caregiving (44.06%). The items with the best correct rate were “People whose Alzheimer's disease is not yet severe can benefit from psychotherapy for depression and anxiety” (96.20%), “A person with Alzheimer's disease becomes increasingly likely to fall down as the disease gets worse” (95.54%), and “Genes can only partially account for the development of Alzheimer's disease” (93.97%), while the poorest responses were “It has been scientifically proven that mental exercise can prevent a person from getting Alzheimer's disease” (11.40%), “If trouble with memory and confused thinking appears suddenly, it is likely due to Alzheimer's disease” (19.79%), and “Tremor or shaking of the hands or arms is a common symptom in people with Alzheimer's disease” (26.87%). Additional details are shown in Supplementary Tables 1, 2.

### Multivariate analysis of the Alzheimer's Disease Knowledge Scale scores of community health service center staff and the scores in different fields

1. Specific assignments of linear regression analysis are shown in Supplementary Table 3. Following univariate linear regression analysis (Supplementary Table 4), multivariate linear regression analysis was conducted, based on the inclusion of three factors, i.e., education, current profession, and working year. The results showed that education had a statistically significant effect on ADKS scores (*b* = 0.412, *t* = 2.433, *P* = 0.015), and education was positively correlated with ADKS scores. The influence of participants' current profession on ADKS scores was statistically significant (*b* = −0.124, *t* = −2.153, *P* = 0.032), and the scores of physicians were higher in this regard. The effect of employment period on ADKS scores was not statistically significant (*b* = −0.017, *t* = −1.629, *P* = 0.104). Additional details are shown in Table 2.
2. Education, profession, gender, employment period, AD knowledge training, and relatives/friends who had dementia or AD were selected to construct a multivariate linear regression analysis. The results showed that education had statistically significant effects on “assessment and diagnosis” (*b* = 0.128, *t* = 2.778, *P* = 0.006) and “caregiving” (*b* = 0.136, *t* = 2.039, *P* = 0.042), and education was positively correlated with the scores above. Profession had statistically significant effects on “risk factors” (*b* = −0.058, *t* = −2.445, *P* = 0.015), and the scores of physicians were higher in this regard. Gender had statistical significance for “risk factors” (*b* = −0.183, *t* = −2.066, *P* = 0.039) and “symptoms” (*b* = −0.204, *t* = −2.612, *P* = 0.009), and male participants had higher scores in this regard. Employment period had statistically significant effects on “risk factors” (*b* = −0.019, *t* = −4.574, *P* < 0.001), “assessment and diagnosis” (*b* = 0.007, *t* = 2.650, *P* = 0.008), “life impact” (*b* = 0.011, *t* = 4.071, *P* < 0.001), and “caregiving” (*b* = −0.017, *t* = −4.164, *P* < 0.001). Relatives/friends with dementia or AD had a statistically significant impact on “symptoms” (*b* = −0.176, *t* = −2.041, *P* = 0.042). Additional details are shown in Table 2.

**Table 2:** Multivariate linear regression analysis.

	**Variate**	***b*** **-value**	***b*** **-value SE**	**B standardized values**	***t*** **-value**	***P*** **-value**
ADKS	Constant	19.177	0.522		36.720	< 0.001
	Education	0.412	0.169	0.089	2.433	0.015
	Profession	-0.124	0.058	−0.077	-2.153	0.032
	Working years	-0.017	0.010	−0.059	-1.629	0.104
Risk factor	Constant	4.746	0.343		13.841	< 0.001
	Profession	-0.058	0.024	−0.088	-2.445	0.015
	Gender	-0.183	0.089	−0.075	-2.066	0.039
	Working years	-0.019	0.004	−0.169	-4.574	< 0.001
Assessment and diagnosis	Constant	2.358	0.228		10.328	< 0.001
	Education	0.128	0.046	0.102	2.778	0.006
	Working years	0.007	0.003	0.099	2.650	0.008
Symptoms	Constant	2.868	0.302		9.497	< 0.001
	Relatives or friends had dementia or AD	-0.176	0.086	−0.074	-2.041	0.042
	Gender	-0.204	0.078	−0.096	-2.612	0.009
Life impact	Constant	2.256	0.216		10.440	< 0.001
	Working years	0.011	0.003	0.152	4.071	< 0.001
Caregiving	Constant	2.030	0.331		6.136	< 0.001
	Education	0.136	0.067	0.074	2.039	0.042
	Working years	-0.017	0.004	−0.154	-4.164	< 0.001

ADKS, Alzheimer's Disease Knowledge Scale; AD, Alzheimer's disease; SE, standard error.

*p*-value was based on multivariate linear regression analysis.

### Comparing the two groups

In total, 261 people who were willing to receive training were randomly divided into two groups according to the institution in which they worked, i.e., an online (119 people) and offline (142 people) training group, respectively. Before conducting the training, there was no statistical difference between the two groups in age, education, professional title, working years, training on AD-related knowledge, or relatives/friends with dementia or AD (*P* > 0.05); there were statistical differences in gender and profession (*P* < 0.05). Additional details are shown in Table 3.

**Table 3:** Sociodemographic characteristics of the two groups.

**Characteristics**	**Offline group [*n* (%)]**	**Online group [*n* (%)]**	***Z*** **-value**	***P*** **-value**
**Gender**			2.956	0.003
Male	51 (35.9%)	23 (19.3%)		
Female	91 (64.1%)	96 (80.7%)		
Age	33.50 (18)^a^	34.00 (10)^a^	0.302	0.762
**Education**			1.742	0.082
Secondary	1 (0.7%)	4 (3.4%)		
Vocational training	32 (22.5%)	34 (28.6%)		
Bachelor	108 (76.1%)	81 (68.1%)		
Master and above	1 (0.7%)	0 (0)		
**Professional title**			1.362	0.173
Junior	60 (42.3%)	57 (47.9%)		
Intermediate	36 (25.4%)	37 (31.1%)		
Associate senior	21 (14.8%)	10 (8.4%)		
Senior	9 (6.3%)	0 (0)		
Others	16 (11.3%)	15 (12.6%)		
**Profession**			2.160	0.031
Physician	80 (56.3%)	44 (37.0%)		
Surgeon	9 (6.3%)	4 (3.4%)		
Nursing	22 (15.5%)	47 (39.5%)		
Pharmacy	6 (4.2%)	10 (8.4%)		
Administration	12 (8.5%)	12 (10.1%)		
Support	3 (2.1%)	2 (1.7%)		
Technician	10 (7.0%)	0 (0)		
Working years	10.00 (19.0)^a^	11.00 (12.0)^a^	0.594	0.552
**Training on AD knowledge**			1.827	0.068
Yes	40 (28.2%)	22 (18.5%)		
No	102 (71.8%)	97 (81.5%)		
**Relatives or friends had dementia or AD**			1.284	0.199
Yes	33 (23.2%)	20 (16.8%)		
No	109 (76.8%)	99 (83.2%)		

a Median (quartile distance).

AD, Alzheimer's disease.

*p*-value was based on rank-sum test.

### The Alzheimer's Disease Knowledge Scale and domain scores of 261 participants

Before training, the average ADKS score of 261 staff was 20.24, with an awareness rate of 67.74%; after training, the average ADKS score was 20.88, with an awareness rate of 69.87%. The ADKS scores (*Z* = 3.903, *P* < 0.001), “treatment and management” (*Z* = 3.498, *P* < 0.001), “life impact” (*Z* = 2.423, *P* = 0.015), “assessment and diagnosis” (*Z* = 2.394, *P* = 0.017), “risk factors” (*Z* = 2.497, *P* = 0.013), and “symptoms” (*Z* = 4.357, *P* < 0.001) aspects showed statistical differences following training. More details are shown in Table 4.

**Table 4:** ADKS content domains and scores of 261 people.

**Items**	**Score range**	**Before training**		**After training**		***Z*** **-value**	***P*** **-value**
		**Score**	**Correct rate**	**Score**	**Correct rate**		
ADKS scores	0–30	20.00 (3)	67.74%	21.00 (2)	69.87%	3.903	<0.001
Treatment and management	0–4	3.00 (1)	83.05%	4.00 (1)	87.64%	3.498	<0.001
Life impact	0–3	2.00 (1)	78.93%	2.00 (1)	74.71%	2.423	0.015
Assessment and diagnosis	0–4	3.00 (1)	68.97%	3.00 (0)	71.84%	2.394	0.017
Risk factors	0–6	4.00 (1)	68.39%	4.00 (1)	70.88%	2.497	0.013
Symptoms	0–4	2.00 (1)	59.39%	3.00 (1)	66.67%	4.357	<0.001
Course	0–4	3.00 (1)	75.67%	3.00 (2)	77.30%	0.995	0.320
Caregiving	0–5	2.00 (1)	47.36%	2.00 (1)	46.59%	0.582	0.561

Scores are described as median (quartile distance).

ADKS, Alzheimer's Disease Knowledge Scale.

*p*-value was based on rank-sum test.

### The Alzheimer's Disease Knowledge Scale scores before and after training

Before training, there were no significant differences in the ADKS scores and awareness rates between the two groups (*Z* = 1.422, *P* = 0.155). After training, there were also no significant differences in the ADKS score and awareness rate between the two groups (*Z* = 1.099, *P* = 0.313). There were statistical differences in ADKS scores and awareness rates between the offline and online training groups before and after training (*Z* = 2.275, *P* = 0.023; *Z* = 3.145, *P* = 0.002). Additional details are shown in Table 5.

**Table 5:** ADKS scores before and after training.

**Group**	**Before training**	**After training**	***Z*** **-value**	***P*** **-value**
	**ADKS scores**	**ADKS scores**		
	**[Median (quartile distance)]**	**[Median (quartile distance)]**		
Offline training group	21.00 (3)	21.00 (4)	2.275	0.023
Online training group	20.00 (2)	22.00 (3)	3.145	0.002
*Z*-value	1.422	1.099		
*P*-value	0.155	0.313		

ADKS, Alzheimer's Disease Knowledge Scale.

*p*-value was based on rank-sum test.

### Feedback information

A total of 111 of 119 participants of the online training group provided feedback information, most of whom agreed or agreed to some extent that “online training was an acceptable form of training,” “the training generally felt good,” “the training would be of great help to their future work,” and “they would also be willing to take part in online training in the future”; the above statements accounted for 77.48, 75.68, 77.48, and 77.48% of the respondents, respectively. Additional details are shown in Supplementary Table 5.

## Discussion

This is the first study to use ADKS to investigate the knowledge of Alzheimer's disease among community health service center staff in Jiaxing, and to conduct training and evaluate the effectiveness of the training. At present, the consultation rate of patients with dementia in China is not high, and most patients live in the community. In order that more patients with dementia can be identified, diagnosed, intervened and treated as soon as possible, the role of CHSC staff is of great importance. Understanding the current level of AD-related knowledge of CHSC staff in this area and their response and effectiveness to training is a favorable reference for designing training and formulating relevant policies in the future.

The current study survey showed that the average ADKS score of 763 community staff members was 19.77, and the awareness rate was only 65.92%. This was similar to the results of studies conducted by Lin et al. (10), Wang et al. (11), and Liu et al. (12) (with scores of 20.41 ± 2.94, 19.7 ± 3.07, and 19.60 ± 2.70, respectively) but significantly lower than the results in research conducted by Smyth et al. (13), Alacreu et al. (14), Zerafa and Scerri (15), and Wang et al. (16) (with scores of 26, 24.4, 21.46 ± 3.41, and 21.42 ± 2.73, respectively); it was also lower than the public awareness rate required by the National Health Commission. This suggests that the AD-related knowledge of community health service center staff in Jiaxing was poor and required urgent improvement, particularly in the “symptoms” and “caregiving” dimensions (the percent accuracy was lower than 60%), which is consistent with the investigative conclusions of Liu et al. (12) and He et al. (17). These two aspects should thus be strengthened in the future training of AD-related knowledge for community health service center staff.

The multivariate regression analysis conducted for this study showed that education had an impact on ADKS scores, “assessment and diagnosis,” and “caregiving” aspects; additionally, a higher educational level yielded higher scores in the above areas, which was consistent with the results of various studies at home and abroad (17, 18). According to the current study survey, most community health service center staff members in Jiaxing had a bachelor's degree, which represented a true reflection of the community's educational background. The process of learning has little to do with academic qualifications. Community health service center service centers should call on their staff to actively participate in training and additional learning, which will help to improve their knowledge and skills in all aspects. The profession aspect affected ADKS scores and “risk factors,” and physicians had a higher score than other professionals (such as nurses) in this regard. Several investigations at home and abroad showed low scores for “risk factors” (13, 14, 16, 18–20), which was also the case in the present study. The correct rate of “risk factors” was only 65.05%, which was much lower than the highest correct rate for “treatment and management” (81.32%). In 2020, the National Health Commission pointed out that community health service centers should increase publicity and education, improve public awareness of mental health, and enhance residents' understanding of prevention and treatment regarding dementia. The staff of medical institution at all levels, offices for the aged, institution for the elderly, and institution combining medical and nursing care should create materials advertising prevention and treatment, based on the characteristics of patients and high-risk groups, thereby giving the public free access to scientific knowledge and resources about dementia (National Health Office Disease Control Letter, 2020, No. 726). Community health service center staff should engage in additional studies of the risk factors of AD/dementia and other related knowledge to be more efficient in the popularization of scientific knowledge and their daily work.

Relatives or friends who suffered from dementia/AD had an impact on “symptoms,” and participants whose relatives/friends suffered from dementia or AD had higher scores. This was consistent with a study conducted by Liu et al. (12). Relatives or friends who suffered from dementia/AD did not affect ADKS scores, which was consistent with the study results presented by Alacreu et al. (14) and Amado and Scerri (21). Therefore, staff members who had friends or relatives with dementia/AD did not have higher ADKS scores, indicating that the ADKS was multi-dimensional and comprehensive. Training on AD knowledge did not affect ADKS scores and was an unanticipated result. Except for the results presented by Zarafa and Scerri (15), this outcome was inconsistent with other studies (13, 17, 18). Considering that previous training did not achieve the anticipated effect, community staff may not have paid significant attention to the training, thereby giving rise to an overall bad effectiveness. Or the training content may not have been regularly strengthened, some aspects thereof had been forgotten. The items with the poorest responses were “It has been scientifically proven that mental exercise can prevent a person from getting Alzheimer's disease” (in fact, mental exercise does help to improve the symptoms of AD, but it can't avoid the occurrence of AD), “If trouble with memory and confused thinking appears suddenly, it is likely due to Alzheimer's disease” (we all know that AD is chronic, a sudden memory disorder always means other acute diseases), and “Tremor or shaking of the hands or arms is a common symptom in people with Alzheimer's disease” (we specialist physicians know that tremor generally refers to Parkinson's disease or idiopathic tremor). The focus of present training was the clinical manifestations of AD, related risk factors, diagnosis, treatment and prognosis, did not include tremors or other related diseases in our department, thus some community health service center staff might answer wrongly. Therefore, we consider that we should further enrich the training content in the future, involving a wider range of knowledge, in order to better help community health service center staff to identify patients with cognitive impairment in the early stage. Also, the sample size should be expanded in future studies to further verify the findings in this context.

The number of years staff members had been employed had an impact on “risk factors,” “assessment and diagnosis,” “life impact,” and “caregiving.” A longer period of employment indicated higher scores in the “assessment and diagnosis” and “life impact” categories but lower scores for “risk factors” and “caregiving.” The period of employment was positively correlated with age. Several foreign studies (18, 19) posited that ADKS scores increased with age; other studies (16, 22) suggested that ADKS scores were higher among younger individuals. Therefore, the effects of age and employment period on ADKS scores in every dimension require further verification.

The effect of gender on ADKS scores and all dimensions included was not determined. This study suggests that gender had an impact on “risk factors” and “symptoms,” and that males had higher scores in this instance. In a study conducted by Alacreu et al. (14), male pharmacists scored lower in the “risk factors” but higher in “assessment and diagnosis,” while male general practitioners scored higher in “risk factors” but lower in “symptoms.” Studies (16, 23) suggested that gender did not influence the above-noted scores. The majority of people in the present study were female and, as such, some bias may have been present; this should be verified by expanding the sample size.

According to the present study, the awareness rate of AD knowledge among community health service center staff in Jiaxing was much lower than the national requirements. Therefore, targeted training is urgently needed to address this. This study showed that the ADKS scores for “treatment and management,” “assessment and diagnosis,” “risk factors,” and “symptoms” among the community health service center staff who volunteered to participate in the training had all been improved after completing the training. The ADKS scores of the offline and online training groups were both improved after the training compared with those before the training, with statistical significance. Accordingly, training indicated obvious importance. After completing the training, however, the awareness rate still did not meet the general requirements. Enhancement in different dimensions was unbalanced, as such, the role of one-time training was relatively limited. Hu et al. (24) showed that early recognition skills training concerning AD could improve the knowledge of doctors about the disease in community health service centers. Additionally, intensive training was better than conventional training and could also improve the AD screening rate to some extent. Zhang et al. (25) applied modular teaching and training to improve the core competencies and nursing service satisfaction of community support work among senior nurses. Chavda et al. (26) conducted a study on medical students practicing in the community, and the results also showed that the effect of modular teaching was better compared with traditional teaching and that such an approach should be more frequently applied to clinical medical positions in the community. The above conclusions have useful reference value for carrying out training on AD-related knowledge among community health service center staff in the future.

Traditional training occurs offline and in person, and the place and time for its delivery are relatively fixed, and interaction during its delivery is relatively strong. The development of the Internet has gradually changed people's work, study and life, with online training has gradually entered people's attention. Particularly considering the outbreak of the novel coronavirus 2019, online teaching is useful for delivering training in a non-crowded (less contagious) environment. While the time and place in which online learning is delivered are highly selectivity, the interaction aspect is relatively poor and lacks supervision and management. In this study, staff members who volunteered to take part in the training were randomly divided into two groups (offline and online training, respectively). Due to the unit random grouping method, there were statistically significant differences between the two groups in terms of gender and current majors before the training, which is a common drawback of the current situation survey. Univariate linear regression analysis showed that gender and profession had no causal relationship with ADKS scores before and after training and, as such, they were not considered confounding factors. There was no significant difference in ADKS scores between the two groups before training and, as a result, the baseline data were comparable. There was no significant difference in ADKS scores between the two groups after training, either, suggesting a lack of significant difference in the training effect between the two methods (online and offline).

An interprofessional team from the Department of Veterans Affairs, South Central Mental Illness Research (Houston, Texas, USA) designed the Program for Advancing Cognitive Disorders Education for Rural Staff to improve clinician competency and comfort when caring for individuals with dementia. Based on an interprofessional needs assessment, the team created six 1-h training modules, all of which are available for free *via* a network platform. A large number of interprofessional healthcare learners, such as nurses, physicians, psychologists, and social workers, had completed the modules with high satisfaction rates (27). Bussotti et al. (28) also showed that online training was comprehensive and had strong potential.

The internet has become an important tool for learning and teaching. Training can be carried out online in the current pandemic environment, thereby reduce personnel gathering, save time, and complete the training task. In the present study, the majority of participants agreed that online training was acceptable. Xu et al. (29) conducted pre-employment training for new nurses using a combined online/offline method, and the results showed that the method could improve the theoretical skills level and satisfaction of new nurses more than using the offline method only. In the current study, the authors only compared the differences between offline and online training. More appropriate and efficient training methods can be further explored in future studies.

The study also has some limitations. Firstly, a cross-sectional design of the present study could not determine the causal relationships, only associations between knowledge and related influence factors. Secondly, because of the stratified cluster sampling in this study, some bias might be present in the sample selection, thus the selected staff population may not reflect community health services centers (CHSCs) in other parts of Zhejiang Province or other parts of China when generalizing the findings. Thirdly, in the questionnaire survey, the offline training group completed hard copies of the survey, while the online training group completed it digitally using a provided link, which may have had a degree of influence. Fourthly, CHSC staff were also not enthusiastic about participating in training on AD knowledge. Fifthly, female participants outnumbered their male counterparts in the study; in the grouping study, the majority of the participants were female, which imbued the research with some limitations.

## Conclusion

1. The staff of community health service centers in Jiaxing had a low awareness rate of AD-related knowledge, particularly in the “symptoms” and “caregiving” dimensions. 2. Education background and occupation are the influencing factors of ADKS score, and those with higher education background and physicians score more, and the years of working is not the influencing factor of ADKS score. 3. One-time training on AD knowledge could improve this shortcoming among community health service center staff to some extent but still failed to meet the national requirements. There were no obvious differences between offline and online training.

Based on a variety of factors, offline, online, or a combination of both can be used to carry out training in the future; in doing so, assurances must be made that the training is repeated and updated, which will help to strengthen the training. This will help to improve the AD-related knowledge and skills of grassroots healthcare staff and provide a solid foundation for the early discovery, early diagnosis, and early treatment of AD in the community.

## Data availability statement

The raw data supporting the conclusions of this article will be made available by the authors, without undue reservation.

## Ethics statement

This study was conducted with approval from the Ethics Committee of the Second Affiliated Hospital of Jiaxing University (No. JXEY-2020JX065). The patients/participants provided their written informed consent to participate in this study.

## Author contributions

WM and XZ: conception and design of the research and obtaining financing. WM, LZ, JT, WD, LQ, and XF: acquisition of data. WM, LZ, JT, and XZ: analysis and interpretation of the data. WM, WD, LQ, and XF: statistical analysis. WM: writing of the manuscript. XZ: critical revision of the manuscript for intellectual content. All authors have read and approved the final draft.

## Footnotes

^1^ http://www.zchsp.cn/index.php/home/news/info.html?id=88&catId=32
