# Supplementary material for: Testing the knowledge of Alzheimer's disease via an intervention study among community health service center staff in Jiaxing, China
Source: Front Public Health. 2023 Jan 27;10:969653. doi: 10.3389/fpubh.2022.969653 (PMC9911520; doi:10.3389/fpubh.2022.969653)
Supplement: Supplementary file 1 [file Table_1.DOC]

Supplementary Table 1: ADKS content domains and scores

| content domains | score range | Correct answers (%) | score[Median (quartile distance)] |
| --- | --- | --- | --- |
| Treatment and management | 0-4 | 81.32 | 3.00 (1) |
| Life impact | 0-3 | 77.76 | 2.00 (1) |
| Course | 0-4 | 75.23 | 3.00 (1) |
| Assessment and Diagnosis | 0-4 | 68.94 | 3.00 (1) |
| Risk factors | 0-6 | 65.05 | 4.00 (2) |
| Symptoms | 0-4 | 57.90 | 2.00 (1) |
| Caregiving | 0-5 | 44.06 | 2.00 (1) |
| Total | 0-30 | 65.92 | 20.00 (4) |

ADKS: Alzheimer’s Disease Knowledge Scale
