# Supplementary material for: Testing the knowledge of Alzheimer's disease via an intervention study among community health service center staff in Jiaxing, China
Source: Front Public Health. 2023 Jan 27;10:969653. doi: 10.3389/fpubh.2022.969653 (PMC9911520; doi:10.3389/fpubh.2022.969653)
Supplement: Supplementary file 2 [file Table_2.DOC]

Supplementary Table 2: Community Health Service Center staff’s AD knowledge measured by the ADKS (by items, n = 763)

| Items | Number of correct answer (%) |
| --- | --- |
| 1. People with Alzheimer’s disease are particularly prone to depression. | 633 (82.96) |
| 2. It has been scientifically proven that mental exercise can prevent a person from getting Alzheimer’s disease. | 87 (11.40) |
| 3. After symptoms of Alzheimer’s disease appear, the average life expectancy is 6 to 12 years. | 574 (75.23) |
| 4. When a person with Alzheimer’s disease becomes agitated, a medical examination might reveal other health problems that caused the agitation. | 662 (86.76) |
| 5. People with Alzheimer’s disease do best with simple, instructions given one step at a time. | 580 (76.02) |
| 6. When people with Alzheimer’s disease begin to have difficulty taking care of themselves, caregivers should take over right away. | 287 (37.61) |
| 7. If a person with Alzheimer’s disease becomes alert and agitated at night, a good strategy is to try to make sure that the person gets plenty of physical activity during the day. | 231 (30.28) |
| 8. In rare cases, people have recovered from Alzheimer’s disease. | 303 (39.71) |
| 9. People whose Alzheimer’s disease is not yet severe can benefit from psychotherapy for depression and anxiety. | 734 (96.20) |
| 10. If trouble with memory and confused thinking appears suddenly, it is likely due to Alzheimer’s disease. | 151 (19.79) |
| 11. Most people with Alzheimer’s disease live in nursing homes. | 445 (58.32) |
| 12. Poor nutrition can make the symptoms of Alzheimer’s disease worse. | 656 (85.98) |
| 13. People in their 30s can have Alzheimer’s disease. | 465 (60.94) |
| 14. A person with Alzheimer’s disease becomes increasingly likely to fall down as the disease gets worse. | 729 (95.54) |
| 15. When people with Alzheimer’s disease repeat the same question or story several times, it is helpful to remind them that they are repeating themselves. | 211 (27.65) |
| 16. Once people have Alzheimer’s disease, they are no longer capable of making informed decisions about their own care. | 372 (48.75) |
| 17. Eventually, a person with Alzheimer’s disease will need 24-h supervision. | 690 (90.43) |
| 18. Having high cholesterol may increase a person’s risk of developing Alzheimer’s disease. | 656 (85.98) |
| 19. Tremor or shaking of the hands or arms is a common symptom in people with Alzheimer’s disease. | 205 (26.87) |
| 20. Symptoms of severe depression can be mistaken for symptoms of Alzheimer’s disease. | 597 (78.24) |
| 21. Alzheimer’s disease is one type of dementia. | 694 (90.96) |
| 22. Trouble handling money or paying bills is a common early symptom of Alzheimer’s disease. | 567 (74.31) |
| 23. One symptom that can occur with Alzheimer’s disease is believing that other people are stealing one’s things. | 567 (74.31) |
| 24. When a person has Alzheimer’s disease, using reminder notes is a crutch that can contribute to decline. | 506 (66.32) |
| 25. Prescription drugs that prevent Alzheimer’s disease are available. | 439 (57.54) |
| 26. Having high blood pressure may increase a person’s risk of developing Alzheimer’s disease. | 614 (80.47) |
| 27. Genes can only partially account for the development of Alzheimer’s disease. | 717 (93.97) |
| 28. It is safe for people with Alzheimer’s disease to drive, as long as they have a companion in the car at all times. | 702 (92.01) |
| 29. Alzheimer’s disease cannot be cured. | 586 (76.80) |
| 30. Most people with Alzheimer’s disease remember recent events better than things that happened in the past. | 428 (56.09) |
