# Supplementary material for: Testing the knowledge of Alzheimer's disease via an intervention study among community health service center staff in Jiaxing, China
Source: Front Public Health. 2023 Jan 27;10:969653. doi: 10.3389/fpubh.2022.969653 (PMC9911520; doi:10.3389/fpubh.2022.969653)
Supplement: Supplementary file 3 [file Table_3.DOC]

Supplementary Table 3: Influence factors analysis assignment

| Factors | Assignment method |
| --- | --- |
| Gender | male=1,female=2 |
| Education | Secondary=1,Vocational training=2,Bachelor=3,Master and above=4 |
| Professional title | Junior=1,Intermediate=2,Associate senior=3,Senior=4,Others=5 |
| Profession | Physician=1,Surgeon=2,Nursing=3,Pharmacy=4,Administration=5,Support=6,Technician=7 |
| Training on AD knowledge | Yes=1,No=2 |
| Reletives or friends had dementia or AD | Yes=1,No=2 |

AD: Alzheimer’s Disease
