# Supplementary material for: Testing the knowledge of Alzheimer's disease via an intervention study among community health service center staff in Jiaxing, China
Source: Front Public Health. 2023 Jan 27;10:969653. doi: 10.3389/fpubh.2022.969653 (PMC9911520; doi:10.3389/fpubh.2022.969653)
Supplement: Supplementary file 4 [file Table_4.DOC]

Supplementary Table 4: Univariate linear regression analysis

| variate | b value | b value SE | B Standardized values | *t* value | *P* value |
| --- | --- | --- | --- | --- | --- |
| Education | 0.469 | 0.168 | 0.101 | 2.798 | 0.005 |
| Profession | -0.134 | 0.058 | -0.084 | -2.313 | 0.021 |
| Working years | -0.022 | 0.010 | -0.077 | -2.134 | 0.033 |

SE: standard error.
