# Supplementary material for: Testing the knowledge of Alzheimer's disease via an intervention study among community health service center staff in Jiaxing, China
Source: Front Public Health. 2023 Jan 27;10:969653. doi: 10.3389/fpubh.2022.969653 (PMC9911520; doi:10.3389/fpubh.2022.969653)
Supplement: Supplementary file 5 [file Table_5.DOC]

Supplementary Table 5: Feedback information from online training group

| Items/ n (%) | Strongly disagree | Disagree | Generally agree | Agree | Quite agree |
| --- | --- | --- | --- | --- | --- |
| Online training is an acceptable form of training | 17 (15.32) | 0 | 8 (7.21) | 44 (39.64) | 42 (37.84) |
| The training feels generally good | 19 (17.12) | 0 | 8 (7.21) | 42 (37.84) | 42 (37.84) |
| The training will be of great help to my future work | 19 (17.12) | 0 | 6 (5.41) | 42 (37.84) | 44 (39.64) |
| I am also willing to take part in online training next time | 18 (16.22) | 0 | 7 (6.31) | 41 (36.94) | 45 (40.54) |
